# MicroRNA-22-3p ameliorates Alzheimer’s disease by targeting SOX9 through the NF-κB signaling pathway in the hippocampus

**DOI:** 10.1186/s12974-022-02548-1

**Published:** 2022-07-12

**Authors:** Pengcheng Xia, Jing Chen, Yingchao Liu, Xiaolin Cui, Cuicui Wang, Shuai Zong, Le Wang, Zhiming Lu

**Affiliations:** 1 Department of Clinical Laboratory Medicine, Shandong Provincial Hospital Affiliated to Shandong First Medical University, Jinan, Shandong China; 2 School of Clinical and Basic Medical Sciences, Shandong First Medical University & Shandong Academy of Medical Sciences, Jinan, Shandong China; 3 School of Medicine, Shandong University, Jinan, Shandong China

**Keywords:** Alzheimer’s disease, miR-22-3p, *Sox9*, NF-κB, Hippocampus

## Abstract

**Background:**

Studies have suggested that many down-regulated miRNAs identified in the brain tissue or serum of Alzheimer’s disease (AD) patients were involved in the formation of senile plaques and neurofibrillary tangles. Specifically, our previous study revealed that microRNA-22-3p (miR-22-3p) was significantly down-regulated in AD patients. However, the molecular mechanism underlying the down-regulation of miR-22-3p has not been comprehensively investigated.

**Methods:**

The ameliorating effect of miR-22-3p on apoptosis of the Aβ-treated HT22 cells was detected by TUNEL staining, flow cytometry, and western blotting. The cognition of mice with stereotaxic injection of agomir or antagomir of miR-22-3p was assessed by Morris water maze test. Pathological changes in the mouse hippocampus were analyzed using hematoxylin and eosin (HE) staining, Nissl staining, and immunohistochemistry. Proteomics analysis was performed to identify the targets of miR-22-3p, which were further validated using dual-luciferase reporter analysis and western blotting analysis.

**Results:**

The miR-22-3p played an important role in ameliorating apoptosis in the Aβ-treated HT22 cells. Increased levels of miR-22-3p in the mouse hippocampus improved the cognition in mice. Although the miR-22-3p did not cause the decrease of neuronal loss in the hippocampus, it reduced the Aβ deposition. Proteomics analysis revealed Sox9 protein as the target of miR-22-3p, which was verified by the luciferase reporter experiments.

**Conclusion:**

Our study showed that miR-22-3p could improve apoptosis and reduce Aβ deposition by acting on Sox9 through the NF-κB signaling pathway to improve the cognition in AD mice. We concluded that miR-22-3p ameliorated AD by targeting Sox9 through the NF-κB signaling pathway in the hippocampus.

**Supplementary Information:**

The online version contains supplementary material available at 10.1186/s12974-022-02548-1.

## Introduction

Alzheimer’s disease (AD) is the most common neurodegenerative disorder in aged people with an increasing incidence with age in the population worldwide [1]. For example, approximately 6.08 million Americans were diagnosed with either clinical AD or mild cognitive impairment due to AD in 2017 and this number is projected to be 15.0 million by 2060 [2]. The best characterized clinical manifestation of AD is its progressive cognitive decline in patients, which appears at the very early stages of AD, in the absence of the two pathological hallmarks, i.e., senile plaques and neurofibrillary tangles [3, 4]. Although the underlying mechanisms involved in these two pathological changes have been well studied, therapeutic strategies targeting these pathologies have not been successful. To date, there are no effective drugs to prevent or treat the cognitive decline in AD patients.

Since the traditional drugs are ineffective in the treatment of AD [5], we focus on the gene therapy agents, which usually consist of (1) vectors or delivery systems containing engineered genetic constructs, and (2) the active ingredients, i.e., DNA, RNA, genetically modified viruses, bacteria, or cells, which can replace, compensate, block, or correct specific genes by introducing foreign genes into target cells or tissues to achieve the purpose of treating and preventing diseases [6].

As one of the common types of genetic medicines, microRNAs (miRNAs) are a group of non-coding small RNAs with a length of about 22 bp that silence target genes at the post-transcriptional level [7]. Current research confirms that miRNAs are involved in many physiological processes and pathological pathways, including embryonic development, tumorigenesis, and cardiac disease [8–10]. Studies have confirmed that in the brain tissue or serum of AD patients, many miRNAs have been identified as down-regulated and thought to be involved in the formation of senile plaques and neurofibrillary tangles [11]. Some brain-enriched miRNAs, such as miR-124, miR-125b, and miR-132 [12–14], have been revealed to show therapeutic effects on AD. In our previous study, we investigated the changes of miRNA levels in serum of AD patients and found that miR-22-3p was significantly down-regulated [15]. Furthermore, studies showed that miR-22-3p regulated the amyloid β (Aβ) deposition in mouse model of AD by targeting mitogen-activated protein kinase 14 [16], while miR-22 regulated the AD silence disease neuroinflammation [17]. To date, the explicit functions of miR-22-3p remain unclear, in particular, due to the lack of transcriptomic and proteomic verifications of miR-22-3p and its mRNA.

Therefore, in order to further investigate the molecular mechanism underlying the regulatory effect of miR-22-3p, we first confirmed the down-regulation of miR-22-3p in brain tissue and blood of AD patients based on the Gene Expression Omnibus (GEO) database (https://www.ncbi.nlm.nih.gov/geo/). Then, the effect of miR-22-3p on apoptosis of the Aβ-treated HT22 cells was detected by TUNEL staining, flow cytometry, and western blotting. The APPswe/PSEN1dD9 (APP/PS1) mice are widely used as animal models of AD [18]. The cognition of mice with stereotaxic injection of agomir or antagomir of miR-22-3p was assessed by Morris water maze test. Pathological changes in the mouse hippocampus were analyzed using hematoxylin and eosin (HE) staining, Nissl staining, and immunohistochemistry. Proteomics analysis was performed to identify the targets of miR-22-3p, and the identified targets were further validated using dual-luciferase reporter analysis and western blotting analysis. The results showed that miR-22-3P could alleviate apoptosis of the Aβ-treated HT22 cells and improve the behavior and Aβ deposition in mice, while no significant effect was observed on neuronal loss. Proteomics analysis revealed the regulatory effects of miR-22-3p on the Sox9 protein, providing a significant potential for the development of new AD treatments.

## Materials and methods

### Data preparation

DIANA-mitED [19] constitutes a novel database for obtaining miRNA abundance estimates through constant analysis of the comprehensive collection of raw small RNA-Seq (sRNA-Seq) datasets. This database contains expression values from over 15,000 analyzed sRNA-Seq human datasets collected from two repositories, including the Sequence Read Archive (SRA) and The Cancer Genome Atlas (TCGA) [19]. Gene expression profiles of AD were downloaded from the GEO database. The dataset of GSE48350 based on GPL570 [HG-U133_Plus_2] Affymetrix Human Genome U133 Plus 2.0 Array contained the microarray data derived from normal controls (aged 20–99 years) and AD cases from 4 brain regions, including hippocampus, entorhinal cortex, superior frontal cortex, and post-central gyrus. Expression levels of synaptic and immune-related genes were assessed to investigate the age-related and AD-related changes as well as the changes of region-specific patterns. A total of 19 samples of AD and 43 samples of normal controls of hippocampus tissues were included in this study. The dataset of GSE157239 [20] based on microarray data in an Affymetrix^®^ miRNA Array, version 4.1 (Santa Clara, CA, USA), commercially available by Thermo Fisher Scientific (Waltham, MA), contained a total of 16 post-mortem cases, including 8 AD patients (Braak stage III or above) and 8 control individuals without neuropathological lesions or neurological signs. The dataset of GSE120584 [21] based on GPL21263-3D-Gene Human miRNA V21_1.0.0 contained a total of 1601 serum samples.

### Reagents and plasmids

The miRNA scramble, miR-22-3p mimic, miR-22-3p inhibitor, miR-22-3p agomir, and miR-22-3p antagomir were obtained from the RIBOBIO Corporation (Guangzhou, China). To construct the luciferase reporter vectors, the 400-bp 3’UTR sequence (extended 200 bp on both sides of the microRNA targeting sequences) was cloned into the pmirGLO located between NheI and XbaI. Mutated vectors were constructed into the same site changing the seed sequences of miRNA from G to A and C to T. The sequences were synthesized by the RIBOBIO Corporation (Guangzhou, China).

### Cell culture

The mouse hippocampal neuronal cell line, i.e., the HT22 cells (Cat. no. 337709; BeNa Culture Collection, Beijing, China), were cultured in Dulbecco’s modified Eagle’s medium (DMEM) containing 10% fetal bovine serum, 1% 100 µg/ml streptomycin, and 100 units/ml penicillin (Invitrogen, Thermo Fisher Scientific, Waltham, USA) in a 5% CO_2_ incubator at 37 °C to provide humidified atmosphere (Thermo Fisher Scientific, Waltham, USA).

### Aβ1-42 oligomers preparation and neuronal precursor cell treatment

Oligomerization and characterization of Aβ1-42 were carried out as previously described [22]. Briefly, Aβ1-42 oligomers (P9001, rPeptide, Beyotime, Beijing, China) were dissolved at a concentration of 1 mg/ml in hexafluoroisopropanol (HFIP; Sigma-Aldrich, Brussels, Belgium). A smooth stream of nitrogen gas was used to evaporate the HFIP. The resulting pellet was resuspended in DMSO with the buffer replaced with Tris–EDTA using a 5 ml HiTrap desalting column (GE Healthcare, Brussels, Belgium) according to the manufacturer’s instructions. The concentration of eluted peptide was determined using the Bradford method (Bio-Rad, California, USA). The eluted peptide was allowed to aggregate for 2 h at room temperature. Before the treatments, the medium was replaced by serum-free DMEM medium. The HT22 cells were incubated in neural maintenance medium containing 5 mM Aβ1-42 or scramble control peptide for 48 h. Each treatment was repeated with three biological replicates.

### Animals and housing conditions

The APP/PS1 mice were purchased from View Solid Biotech (Beijing, China). All of the APP/PS1 mice were male and 9 months old [14]. The mice were bred and housed in the Animal Facility of Shandong Provincial Hospital affiliated to Shandong University with a 12 h/12 h light/dark cycle and ad libitum access to food and water. All mice were euthanized with an overdose of anesthetics (pentobarbital sodium) and then perfused transcardially with saline for biochemical analysis and with 4% paraformaldehyde for histological analysis. All experimental procedures were approved by the Animal Care and Use Committee of Shandong Provincial Hospital affiliated to Shandong University and were conducted following the institutional guidelines.

### Brain stereotactic injection

The mice were randomly assigned to each of the three groups, i.e., the control group, the agomir group, and the antagomir group. Each group contained a total of six animals, three used for proteomics analysis and three for morphological staining. MiRNAs injections into the dentate gyrus. For stereotactic injections, the miRNAs were infused into the hemispheric right dentate gyrus (anteroposterior: − 2.1 mm from Bregma; mediolateral: 1.9 mm; dorsoventral: 2.2 mm) [23].A total of 2.0 µL antagomir solution (50 μM) was injected into the hippocampal region of each mouse using a small high precision brain stereotaxic instrument (SA-100, Yuyan, Shanghai, China) with the injection speed of 0.2 μL/min. After injection, the needle was retained for 2 min to ensure the drug absorption before the needle was withdrawn slowly. The mice in the control group were injected with the same volume of PBS in the hippocampal regions. In two weeks, the mice were then subjected to a series of behavioral experiments. The brain samples were collected with perfusion after the behavioral experiments.

### Cell apoptosis assay

Cell apoptosis was detected using Annexin V/7-aminoactinomycin D (7-AAD) staining. Briefly, approximately 5 × 10^5^ cells were resuspended in 100 μL binding buffer and stained with 2.5 μL PE-Annexin V and 2.5 μL 7-AAD at 37 °C in darkness for 15 min, and then, 400 μL binding buffer was added. The cells were analyzed by flow cytometry using a fluorescence-activated cell sorting cytometer (BD Biosciences, San Jose, USA).

### Cell proliferation assay

Cell proliferation was assessed by CCK-8 assay (Dojindo, Kumamoto, Japan). In brief, cells were seeded into 96-well plates at a density of 5 × 10^3^ cells/well and serum-starved for 24 h, followed by incubation with the indicated treatment concentration and stimulation for 48 h. Subsequently, 10 µl of CCK-8 reagent was added to each well and incubated for 3 h at 37 °C. OD450 absorbance was measured using a microplate reader.

### Reverse transcription quantitative polymerase chain reaction (RT-qPCR)

The primer sequences were synthetized by RIBOBIO Corporation (Guangzhou, China). Trizol (Thermo Fisher Scientific Inc., MA, USA) method was employed to extract the total RNA in the cells in each group, and the concentration and purity of the RNA were measured. Samples were reacted in an Eppendorf PCR amplifier and the PCR amplification was conducted by SYBR^®^ Premix Ex Taq (Accurate Biotechnology Co., Ltd. Hunan, China) and a real-time quantitative PCR amplifier (Applied Biosystems QuantStudio 5, ABI Company, Oyster Bay, NY), according to the directions of PrimeScript RT reagent Kit with gDNA Eraser (Accurate Biotechnology Co., Ltd. Hunan, China). U6 (F: 5′-CGCTTCGGCAGCACATATAC-3′; R: 5′-TTCACGAATTTGCGTGTCAT-3′) was, respectively, used as the internal reference of miR-22-3p. The data were analyzed by 2^−ΔΔCT^ method.

### Western blot analysis

The total protein of cells or tissues under different treatments was extracted by the radioimmunoprecipitation assay (RIPA) (P0013, Beyotime Biotechnology, Shanghai, China) lysis buffer containing protease inhibitor (PI) (ST506, Beyotime Biotechnology, Shanghai, China) and then centrifugation. The supernatant was collected and the concentration was measured by the bicinchoninic acid (BCA) kit (PC0020, Solarbio, Beijing, China). An equal amount of protein was denatured and separated by 8%, 10%, or 12% SDS-PAGE electrophoresis, and transferred to polyvinylidene fluoride membranes (PVDF) (ISEQ00010, Merck KGaA, Darmstadt, Germany). The membranes were blocked in TBS containing 0.05% tween20 (TBST) with either 5% BSA or defatted milk and incubated with the primary antibodies in TBST with either 5% BSA or defatted milk overnight at 4 °C according to the manufacturer’s protocol. Then, the samples were incubated with second antibodies for 1 h at room temperature after washed thrice with TBST. Finally, the immunoblots were detected using an ECL kit (WBKLS0100, Millipore, Massachusetts, USA). The primary antibodies used included anti-Bcl-2 antibody (1:1000, ab196495, RRID: AB_2783814, Abcam, Massachusetts, USA), anti-Bax antibody (1:1000, ab32503, RRID: AB_725631, Abcam, Massachusetts, USA), anti-Sox9 antibody (1:1000, ab185966, RRID: AB_2728660, Abcam, Massachusetts, USA), anti-NF-kb p65 antibody (1:1000, ab32536, RRID:AB_776751, Abcam, Massachusetts, USA), and anti-β-actin (1:1000, Ta-09, RRID:AB_2636897, ZSGB-BIO, Beijing, China; 1:1000, GB11001, RRID:AB_2801259, Servicbio, Wuhan, China).

### TUNEL staining

TUNEL kit (Beyotime Biotechnology, Shanghai, China) was used to detect the apoptotic level of different groups of cells in vitro according to the manufacturer’s instructions. The results were visualized by a Leica TCS SP8 confocal fluorescence microscope (Leica Microsystems, Biberach, Germany).

### Morris water maze test

Morris water maze test each containing the spatial acquisition (5 days) and probe trial (1 day) was performed on the mice to evaluate the AD-induced spatial learning deficits as previously described [24]. In the spatial acquisition, each mouse was trained 4 times on training days 1–5 with a 20-min interval. In each trial, the mouse that found the round platform (10 cm in diameter and submerged 2 cm under the water) of the target quadrant in a circular metal tank (180 cm in diameter) was allowed to stay on platform for 10 s; each training time did not exceed 2 min. The swimming time (i.e., the escape latency measured in s) and path (distance measured in cm) to reach platform were recorded using an overhead camera with a computerized tracking system (Noldus EthoVision XT 10.0 software). In the probe trial, the mouse was placed in the opposite side of the target quadrant for 1-min exploration training after removing the platform. The time that a mouse spent (% time) and the number of crossovers in the target quadrant were recorded with the computerized tracking system.

### Hematoxylin and eosin staining

The brains of mice were collected and fixed in 4% formaldehyde solution overnight at 4 °C. The paraffin-embedded tissues were sectioned (5 μm thickness) after dehydration and vitrification. The tissue sections were deparaffined with xylene, rehydrated with an ethanol gradient, and stained with HE (Solarbio Science & Technology Co., Ltd., Beijing, China) in proper order. The histopathological changes of the hippocampus were visualized with an optical microscope (Olympus Optical Co. Ltd., Tokyo, Japan) at 400 × magnification.

### Nissl staining

The brain sections were dewaxed, rehydrated, and microwaved in 0.01 M sodium citrate buffer for 5 min. The sections were then cooled to room temperature and washed with PBS three times. After being stained with cresyl violet (Solarbio Science & Technology Co., Ltd., Beijing, China), the sections were dehydrated with 95% ethanol for 5 min, 100% ethanol for 10 min, and xylene for 10 min, and fixed using mounting media. The histopathological changes of the hippocampus were visualized with an optical microscope (Olympus Optical Co. Ltd., Tokyo, Japan) at 400 × magnification.

### Immunohistochemistry

All paraffin‐embedded sections were dewaxed with xylene and dehydrated with an ethanol gradient. The protein expression of Aβ in brain tissues was detected by implementing a two‐step method (PV‐900). The antibody dilution was added as normal control (NC). The sections were washed with 0.1 mol/L PBS for 3 min thrice and then incubated with 3% peroxidase. Next, the sections were incubated with 50 μL of nonimmune goat serum for 30 min. Subsequently, the sections were probed with the primary antibody, i.e., the Aβ rabbit polyclonal antibody (ab126649, RRID: AB_2818982, 1:5000, Abcam, Massachusetts, USA), overnight at 4 °C, the polymerase adjuvant at room temperature for 20 min, and then the horseradish peroxidase (HRP)‐labeled secondary antibody (Beijing Bioss Biotechnology Co., Ltd., Beijing, China) at room temperature for 30 min. The sections were developed using diaminobenzidine (DAB), counterstained with hematoxylin, and mounted for observation. The cells presenting a brownish‐stained cytoplasm were identified as Aβ‐positive. Five fields of view were randomly selected on each section for observation under an optical microscope.

### Proteomics analysis

1. Experimental procedures
  i. TCA/acetone precipitation and SDT Lysis [25]. The samples were first frozen in liquid nitrogen and then ground with a pestle and mortar to powder. Then, 5 times volume of TCA/acetone (1:9) was added to the powder and mixed by vortex. The mixture was placed at – 20 °C for 4 h and centrifuged at 6000*g* for 40 min at 4 °C. The supernatant was discarded. The pre-cooled acetone was added and washed three times. The precipitation was air dried. Then, 30 times volume of SDT buffer was added to 20–30 mg powder, mixed, and boiled for 5 min. The lysate was sonicated and then boiled for 15 min. After centrifugation at 14,000*g* for 40 min, the supernatant was filtered with 0.22-µm filters. The filtrate was quantified with the BCA Protein Assay Kit (P0012, Beyotime Biotechnology, Shanghai, China). The sample was stored at − 20 °C for further use.
  ii. Homogenation and SDT lysis [26]. SDT buffer was added to the sample, and the mixture was transferred to 2-mL tubes with the same amount of quartz sand. The lysate was homogenized by MP Fastprep-24 Automated Homogenizer (6.0 M/S, 30 s, twice). The homogenate was sonicated and then boiled for 15 min. After centrifugation at 14,000*g* for 40 min, the supernatant was filtered with 0.22-µm filters. The filtrate was quantified with the BCA Protein Assay Kit (P0012, Beyotime Biotechnology, Shanghai, China). The sample was stored at − 20 °C for further use.
  iii. Measurement of protein concentration [27]. SDT buffer was added to the sample. The lysate was sonicated and then boiled for 15 min. After centrifugation at 14,000*g* for 40 min, the supernatant was quantified with the BCA Protein Assay Kit (P0012, Beyotime Biotechnology, Shanghai, China). The sample was stored at − 20 °C for further use.
  iv. Immunoaffinity depletion of serum high-abundance proteins. The most abundant proteins of serum pools were depleted using Agilent Mouse 3 Multiple Affinity Removal System Column (Agilent Technologies) following the manufacturer’s protocol [28]. The Mouse 3 column was applied for mouse. The 10-kDa ultrafiltration tube (Sartorius) was used for desalination and concentration of low-abundance components. One volume of SDT buffer was added, boiled for 15 min, and centrifuged at 14,000*g* for 20 min. The supernatant was quantified with the BCA Protein Assay Kit (P0012, Beyotime Biotechnology, Shanghai, China). The sample was stored at − 20 °C for further use.
2. SDS-PAGE separation
  A total of 20 µg of proteins of each sample were mixed with 6X loading buffer and boiled for 5 min. The proteins were separated on 12.5% SDS-PAGE gel. Protein bands were visualized by Coomassie Blue R-250 staining.
3. Filter-aided sample preparation (FASP digestion)
  A total of 200 μg of proteins of each sample were incorporated into 30 μl SDT buffer (4% SDS, 100 mM DTT, and 150 mM Tris–HCl with pH 8.0). The detergent, DTT, and other low-molecular-weight components were removed using UA buffer (8 M Urea and 150 mM Tris–HCl with pH 8.5) by repeated ultrafiltration (Sartorius, 30 kD). Then, a total of 100 μL iodoacetamide (100 mM IAA in UA buffer) was added to block the reduced cysteine residues with the samples incubated for 30 min in darkness. The filters were washed with 100 μL UA buffer thrice and then 100 μL 0.1 M TEAB buffer twice. Finally, the protein suspensions were digested with 4 μg trypsin (Promega) in 40 μL 0.1 M TEAB buffer overnight at 37 °C, and the resulting peptides were collected as a filtrate. The peptide content was estimated by UV light spectral density at 280 nm using an extinction coefficient of 1.1 of 0.1% (g/l) solution that was calculated based on the frequency of tryptophan and tyrosine in vertebrate proteins.
4. TMT labeling
  A total of 100 μg peptide mixture of each sample was labeled using TMT reagent according to the manufacturer’s instructions (Thermo Fisher Scientific).
5. Peptide fractionation with reversed phase (RP) chromatography
  TMT-labeled peptides were fractionated by RP chromatography using the Agilent 1260 infinity II HPLC. The peptide mixture was diluted with buffer A (10 mM HCOONH4 and 5% ACN with pH 10.0) and loaded onto a XBridge Peptide BEH C18 column, 130 Å, 5 µm, 4.6 mm × 100 mm. The peptides were eluted at a flow rate of 1 ml/min with a gradient of 0–7% buffer B (10 mM HCOONH4 and 85% ACN with pH 10.0) for 5 min, 7–40% buffer B during 5–40 min, 40–100% buffer B during 45–50 min, and 100% buffer B during 50–65 min. The elution was monitored at 214 nm based on the UV light trace, and fractions were collected every 1 min during 5–50 min. The collected fractions were combined into 10 fractions and dried via vacuum centrifugation at 45 °C.
6. Mass spectrometry analysis based on Easy nLC
  Each fraction was injected for nanoLC–MS/MS analysis. The peptide mixture was loaded onto the C18-reversed phase analytical column (Thermo Fisher Scientific, Acclaim PepMap RSLC 50 µm × 15 cm, nano viper, P/N164943) in buffer A (0.1% formic acid) and separated with a linear gradient of buffer B (80% acetonitrile and 0.1% formic acid) at a flow rate of 300 nl/min. The linear gradient was determined as follows:
  i. 1-h gradient: 6% buffer B for 3 min, 6–28% buffer B for 42 min, 28–38% buffer B for 5 min, 38–100% buffer B for 5 min, and kept in 100% buffer B for 5 min.
  ii. 1.5-h gradient: 6% buffer B for 5 min, 6–28% buffer B for 63 min, 28–38% buffer B for 10 min, 38–100% buffer B for 7 min, and kept in 100% buffer B for 5 min.
7. LC–MS/MS analysis
  LC–MS/MS analysis was performed on a Q Exactive mass spectrometer (Thermo Fisher Scientific) that was coupled to Easy nLC (Thermo Fisher Scientific) for 60/90 min. The mass spectrometer was operated in positive ion mode. MS data were acquired using a data-dependent top10 method dynamically choosing the most abundant precursor ions from the survey scan (350–1800 m/z) for HCD fragmentation. Survey scans were acquired at a resolution of 70,000 at *m*/*z* 200 with an AGC target of 3e6 and a maxIT of 50 ms. MS2 scans were acquired at a resolution of 35,000 for HCD spectra at *m*/*z* 200 with an AGC target of 2e5 and a maxIT of 45 ms, with the isolation width set to 2 *m*/*z*. Only ions with a charge state between 2 and 6 with a minimum intensity of 2e3 were selected for fragmentation. Dynamic exclusion for selected ions was set to 30 s. Normalized collision energy was set to 30 eV.
8. Data analysis
  MS/MS raw files were processed using MASCOT engine (Matrix Science, London, UK; version 2.6) embedded into Proteome Discoverer 2.2, and searched against the database (Uniprot_MusMusculus_17056_20210125). The search parameters included trypsin as the enzyme used to generate peptides with a maximum of 2 missed cleavages permitted. A precursor mass tolerance of 10 ppm was specified and 0.05 Da tolerance for MS2 fragments. Except for TMT labels, carbamidomethyl (C) was set as a fixed modification. Variable modifications included oxidation (M) and acetyl (protein N-term). A peptide and protein false discovery rate (FDR) of 1% was enforced using a reverse database search strategy. Proteins with fold change > 1.2 and p value (Student’s *t* test) < 0.05 were considered as differentially expressed proteins (DEPs).
9. Bioinformatics analysis
  Gene Ontology (GO) annotation. First, all protein sequences were aligned to the database downloaded from NCBI (ncbi-blast-2.2.28 + -win32.exe) and only the sequences in top 10 and E-value ≤ 1e-3 were kept. Second, the GO terms (database version: go_201504.obo) of the sequence with top Bit-Score by Blast2GO were selected. Then, the annotation from GO terms to proteins was completed by Blast2GO Command Line. After the primary annotation, the InterProScan was used to search the EBI database by motif and then the functional information of motif was added to proteins annotated to improve the annotation. Further improvement of annotation and connection between GO terms were carried out by ANNEX. Fisher's exact test was used to enrich GO terms by comparing the number of DEPs and the total number of proteins correlated to GO terms.

*KEGG pathway annotation*. KEGG pathway analysis was performed using KEGG database. Fisher’s exact test were used to identify the significantly enriched pathways by comparing the number of DEPs and the total number of proteins correlated to pathways.

### Gene set enrichment analysis (GSEA)

Th AD patients were differentiated into two groups based on the expression level of *Sox9*, the bottom half as the low-expression group and the top half the high-expression group. The GSEA based on these two groups was performed by applying signal pathway differences [29]. Molecular Signature Database (MSigDB, v7.0) provided the background gene sets required for this study. Annotated gene sets were used to distinguish subtypes by the identified differentially expressed genes (DEGs) [30]. The consistency *P*-value for each gene set was computed, and the gene sets with *P*-values less than 0.05 were considered as significantly enriched, which were subsequently ranked. The association between disease type and biological processes was analyzed using the GSEA.

### Dual-luciferase reporter assay

RNAhybrid is a tool commonly used for finding the minimum free energy hybridization between a long and a short RNA. The hybridization is performed in a domain mode, i.e., the short sequence is hybridized to the best fitting part of the long one. The tool is primarily used for microRNA target prediction [31]. 293 T cells in their logarithmic growth phase were seeded in the 96-well plates with 1.0 × 10^4^ cells per well, with a total volume of 100 μL per well, and cultured in incubator at 37 °C for 24 h. To dilute miRNA mimics or non-target control, 5 μL OPTI-MEM medium was added. The target gene 3'UTR dual reporter gene vector or mutation vector were diluted in the same way, and dilute 0.25 μL Lipo6000TM transfection reagent in 5 μL OPTI-MEM medium for 5 min, mix and shake gently, and let stand for 5 min. Before adding plasmids and mimics to cells, first add 90 μL of medium to each well, then add 10 μL of the above mixture, to make the final volume of each well to 100 μL. The transfection concentration of mimics was 50 nM with 50 ng plasmid per well. Three replicate wells were set in each group, and the culture medium was replaced after 6 h of transfection. In 48 h after transfection, the medium was aspirated, added with 1 × PBS at 35 μL/well and luciferase reagent 35 μL/well, shaken for 10 min, transferred to LUMITRAC™200 96-well white cell culture plate to measure the fluorescence value.

### Statistical analysis

GraphPad Prism (version 8.0.0) was utilized to perform the statistical analysis. The normality test and homogeneity of variance test were performed on data extracted from GEO database. Data were presented as means ± standard error of the mean (SEM). All the in vitro experiments were executed in triplicates for three independent times. Student’s t tests were performed to compare the means between two groups, whereas the one/two-way ANOVA was used to compare the mean of three or more groups. The data were considered statistically significant with *P* < 0.05 (*), *P* < 0.01 (**), and *P* < 0.005 (***), respectively, while “ns” indicated nonsignificant difference.

## Results

### Reduced level of miR-22-3p in AD patients and its association with AD

**Fig. 1:**
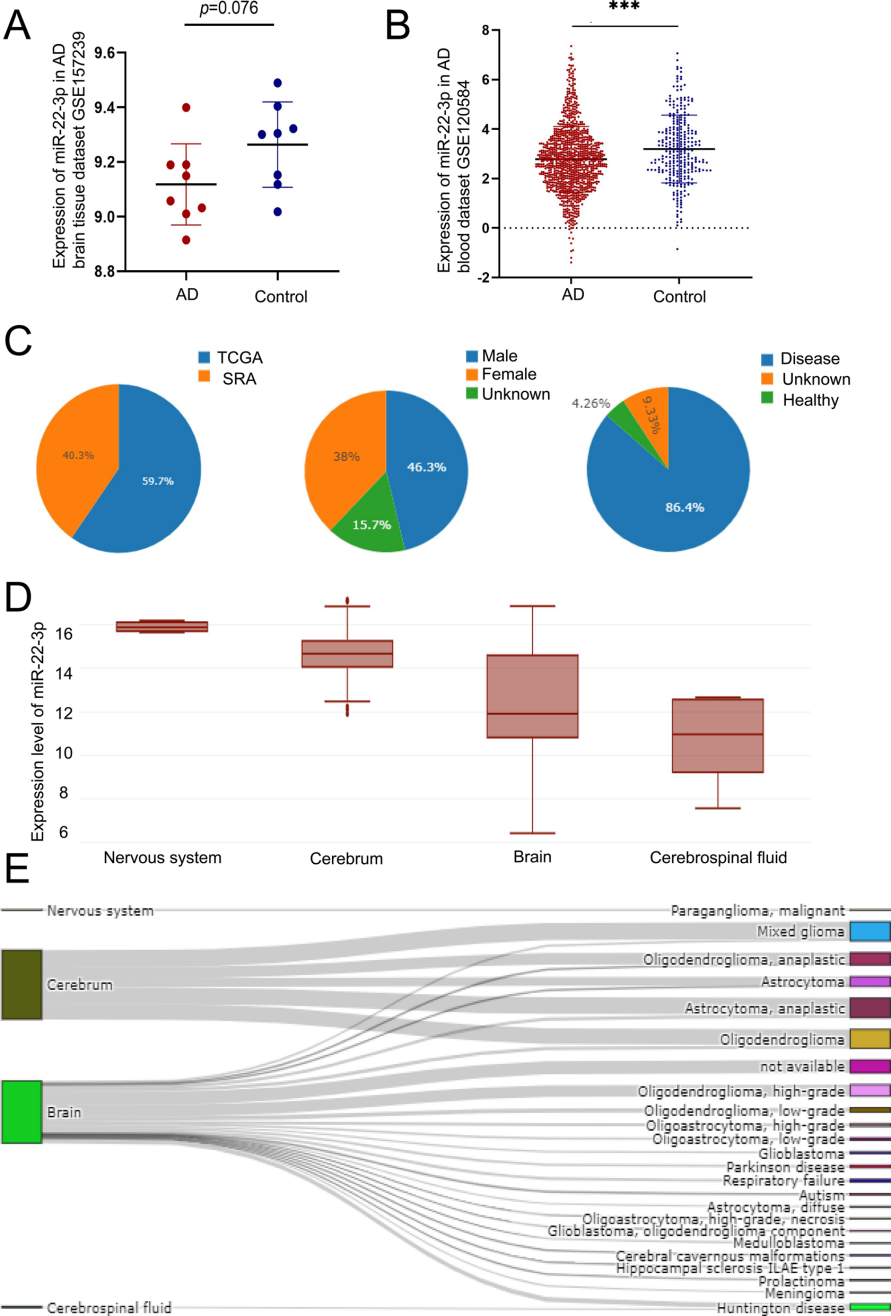
Expression of miR-22-3p in GEO, TCGA, and SRA databases and its relationship with various types of diseases. **A** In the AD brain tissue dataset GSE157239, the expression level of miR-22-3p in AD patients is lower than that in controls. **B** In the AD blood dataset GSE120584, the expression level of miR-22-3p in AD patients is lower than that in controls. **C** Sex and composition ratios of AD patients and healthy individuals in the TCGA and SRA databases. **D** Expression levels of miR-22-3p in different tissues. **E** Association of miR-22-3p with various types of disease. ***P < 0.005

In order to explore the molecular functions of miR-22-3p, the expression levels of miR-22-3p were investigated based on the data in the DIANA-mitED database. The results revealed variations in the expression levels of miR-22-3p in the nervous system, cerebrum, brain, and cerebrospinal fluid. The Sankey diagram further showed that miR-22-3p was associated with many neurological tumor diseases, such as paraganglioma, mixed glioma, and astrocytoma, and other non-tumor diseases, e.g., Parkinson disease, autism, hippocampal sclerosis, ILAE type-1, and Huntington disease (Fig. 1C–E; Additional file 5: Table S3). Then, the expression levels of miR-22-3p in the brain tissue and blood of AD patients were analyzed through the GSE157239 and GSE120584 datasets derived from the GEO database. The results showed that the expression levels of miR-22-3p were decreased in the brain tissue and blood of AD patients (Fig. 1A, B; Additional file 3: Table S1 and Additional file 4: Table S2).

### Ameliorating effects of miR-22-3p on apoptosis in a cellular model of AD

**Fig. 2:**
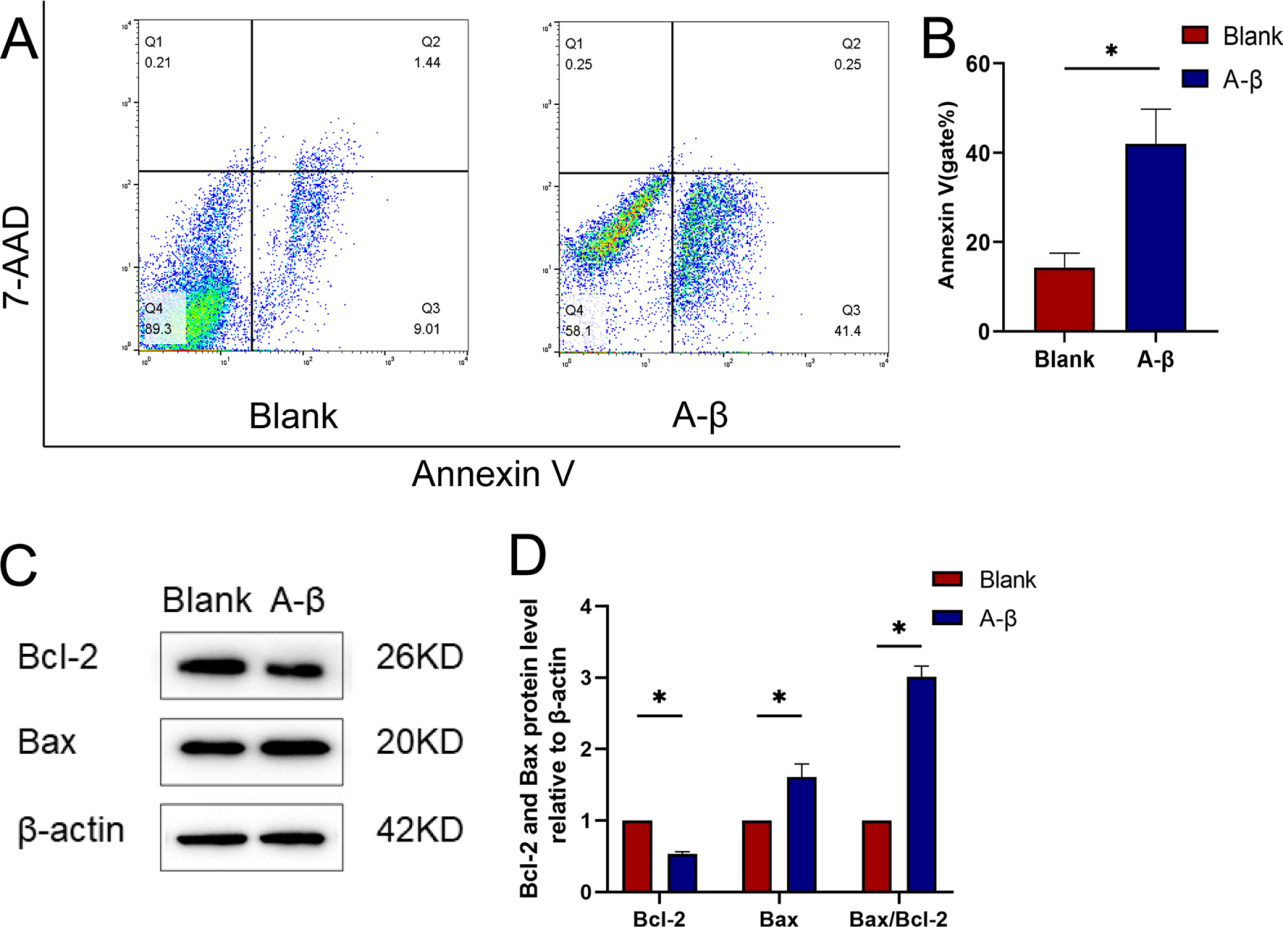
Validation of AD cell model. **A** Flow cytometry analysis reveals the increase of apoptotic cells caused by Aβ. **B** Statistical plot of flow cytometry analysis. **C** Western blot analysis confirms the decreased expression of the anti-apoptotic protein Bcl-2 and the increased expression in the apoptotic protein Bax. **D** Statistical plot of western blot analysis. *P < 0.05, n = 3

**Fig. 3:**
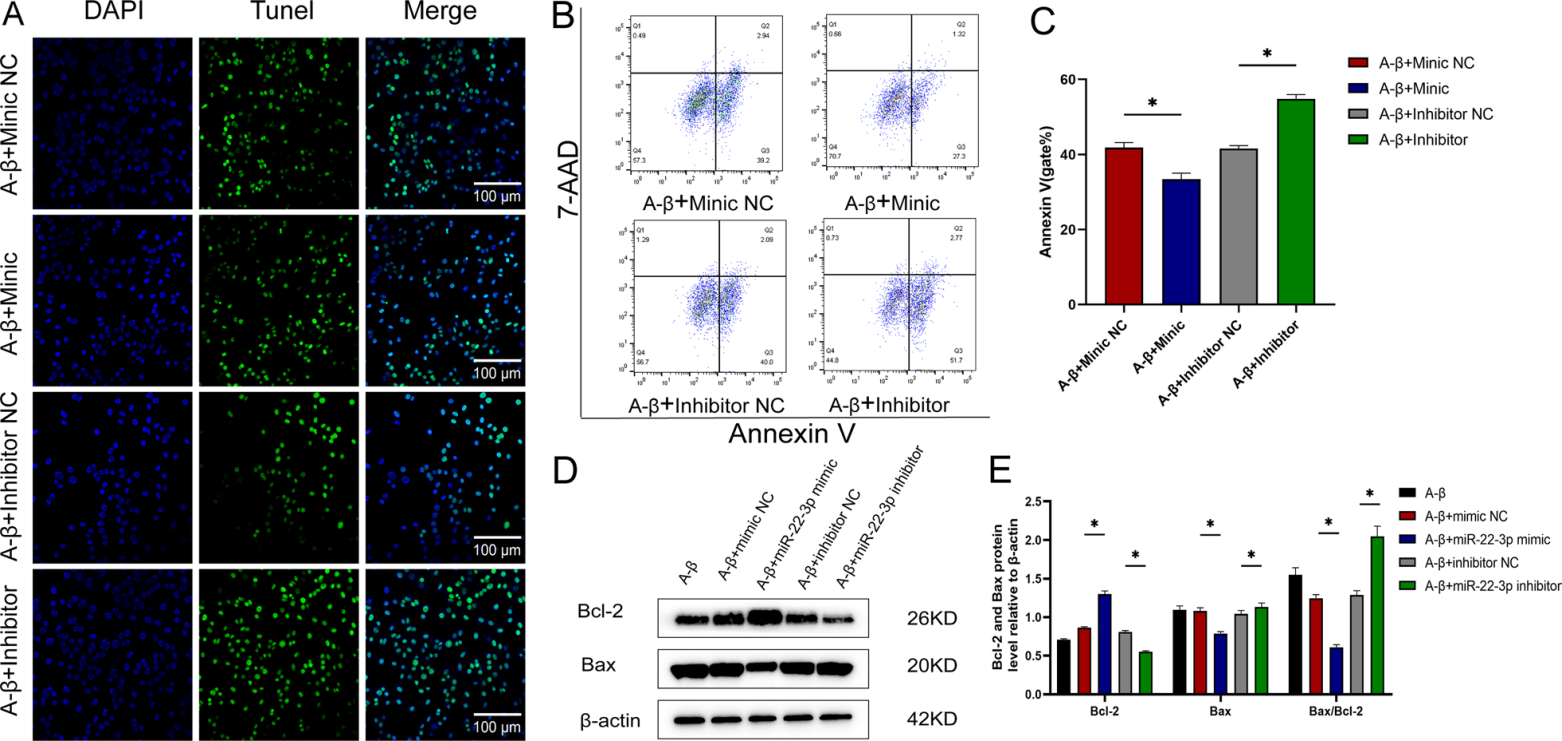
Effect of miR-22-3p on AD cell model. **A** TUNEL assay confirms that the increased level of miR-22-3p improves apoptosis. **B** Flow cytometry analysis confirms that the elevated level of miR-22-3p improves apoptosis. **C** Statistical graph of flow cytometry results. **D** Western blot analysis confirms that miR-22-3p decreases the expression of anti-apoptotic protein Bcl-2 and increases the expression of apoptotic protein Bax. **E** Statistical plot of western blot analysis. *P < 0.05, n = 3

In this study, the mouse hippocampal neuron cell line HT22 was used as a cell model, while the Aβ-induced cells were used to construct a cell model of AD. Studies have shown that Aβ caused the changes in apoptosis-related phenotypes [32]. After the treatment, the apoptosis rate of cells was significantly increased, while the expression of anti-apoptotic protein Bcl-2 was decreased and the expression of pro-apoptotic protein Bax was increased (Fig. 2; Additional file 6: Table S4 and Additional file 7: Table S5). Before performing the experiments, the stability and toxicity of the miRNAs were verified (Additional file 2: Fig. S2), when the concentration of miRNAs was 200 nM, it still did not damage the cells. By changing the expression of miR-22-3p in AD cell model by mimics and inhibitors of miR-22-3p, the results of TUNEL staining and flow cytometry analyses showed that when the level of miR-22-3p was increased, the apoptotic cells were significantly decreased (Fig. 3A–C; Additional file 8: Table S6). Similarly, when the level of miR-22-3p was increased, the expression of anti-apoptotic protein Bcl-2 was increased and the expression of pro-apoptotic protein Bax was decreased (Fig. 3D, E; Additional file 9: Table S7).

### Improved cognition and Aβ deposition by miR-22-3p in AD mice

**Fig. 4:**
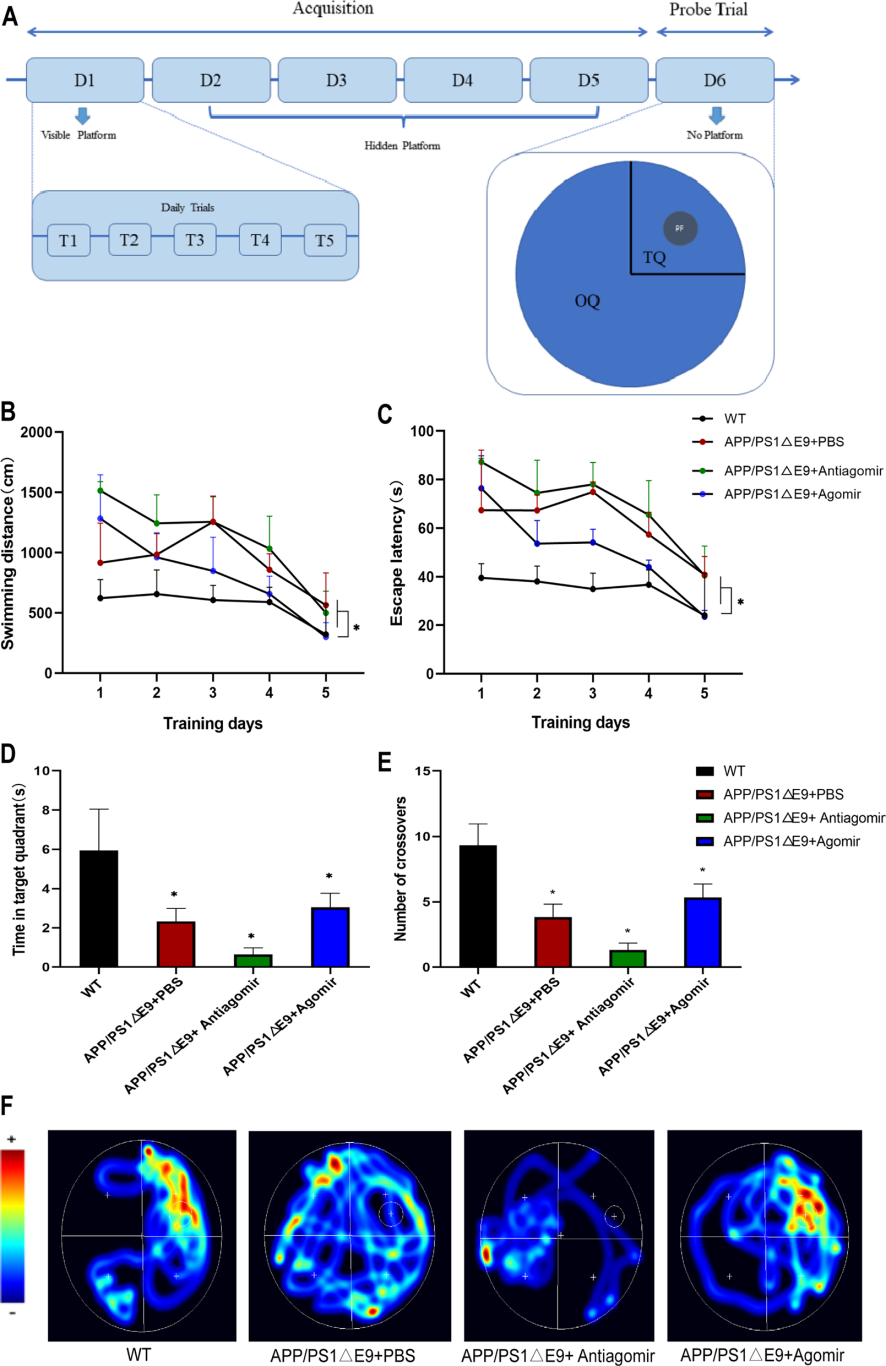
Results of the Morris water maze experiments on mice. **A** Schematic diagram of the water maze experiment. **B** Statistical results of swimming distance. **C** Statistics of escape latency. **D** Statistics of time spent in target quadrant. **E** Statistics of number of crossovers. **F** Heat map of mouse movement trajectories. *P < 0.05

**Fig. 5:**
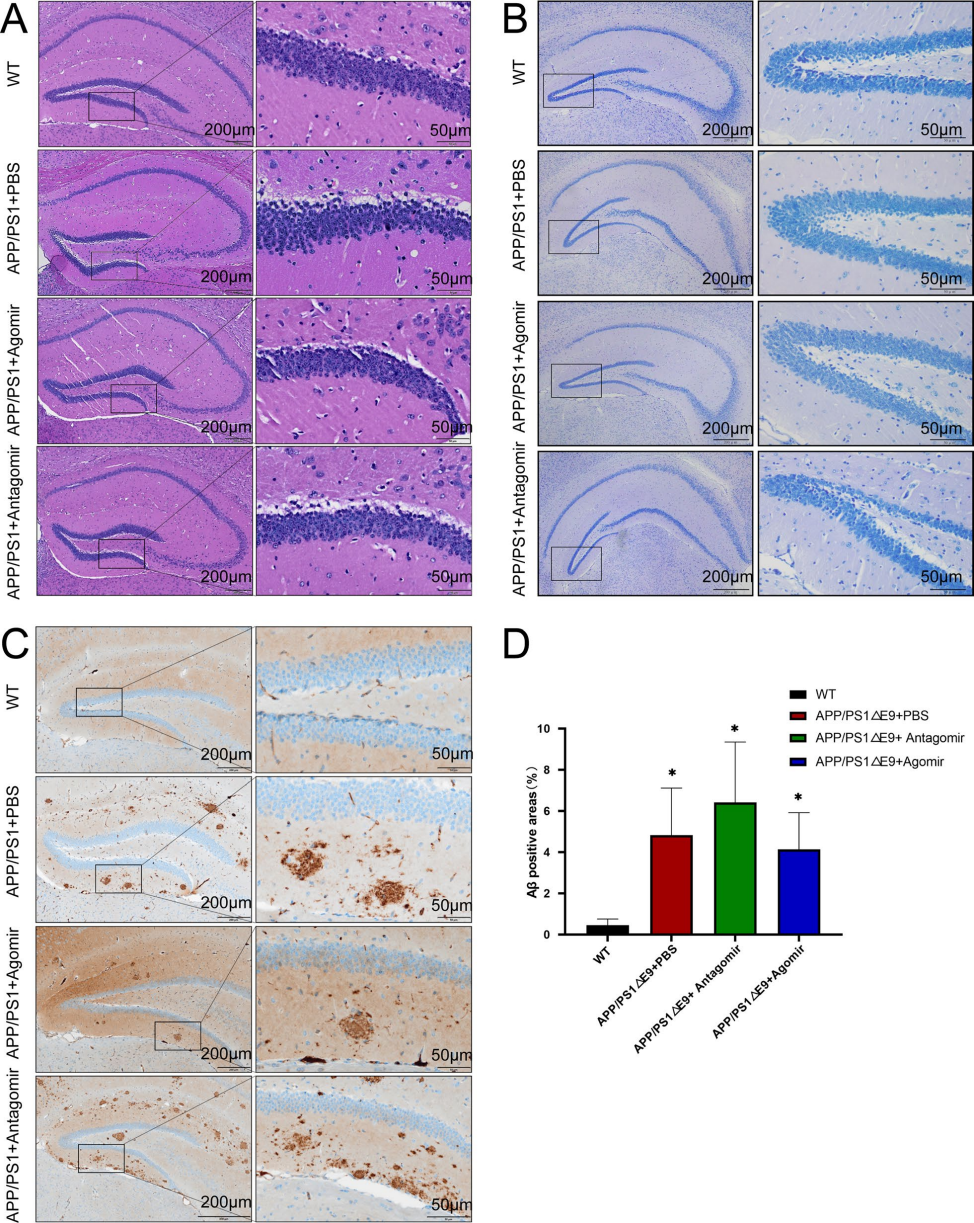
Results of HE (**A**), Nissl (**B**), and immunohistochemical (**C**) stainings and the statistics of immunohistochemical staining (**D**) of Aβ. *P < 0.05, n = 3

The wild C57 mice were used as the normal controls, the APP/PS1 mice as the AD models, the PBS-treated mice as blank controls, the antagomir-treated mice as the low-expression group, and the agomir-treated mice as the high-expression group. Two weeks after the brain stereotactic injection, the Morris water maze tests were performed on mice. The results showed that when the level of miR-22-3p was elevated, the swimming distance and escape latency of mice were significantly reduced, reaching the levels similar to those of C57 mice. Similar patterns were also revealed for the number of crossovers and the amount of time spent in target quadrant. The quadrants were all elevated and these behavioral changes were visualized in the heatmap (Fig. 4; Additional file 10: Table S8). Once the behavioral changes were identified, the morphological examination of the mouse hippocampus was performed. The results of HE staining and Nissl staining revealed no evident loss of neuronal cells (Fig. 5; Additional file 11: Table S9). Then, the Aβ was stained by immunohistochemistry to show that when the level of miR-22-3p was elevated, the deposition of Aβ was significantly reduced.

### MiR-22-3p regulates the protein expressions in the hippocampus of AD mice

**Fig. 6:**
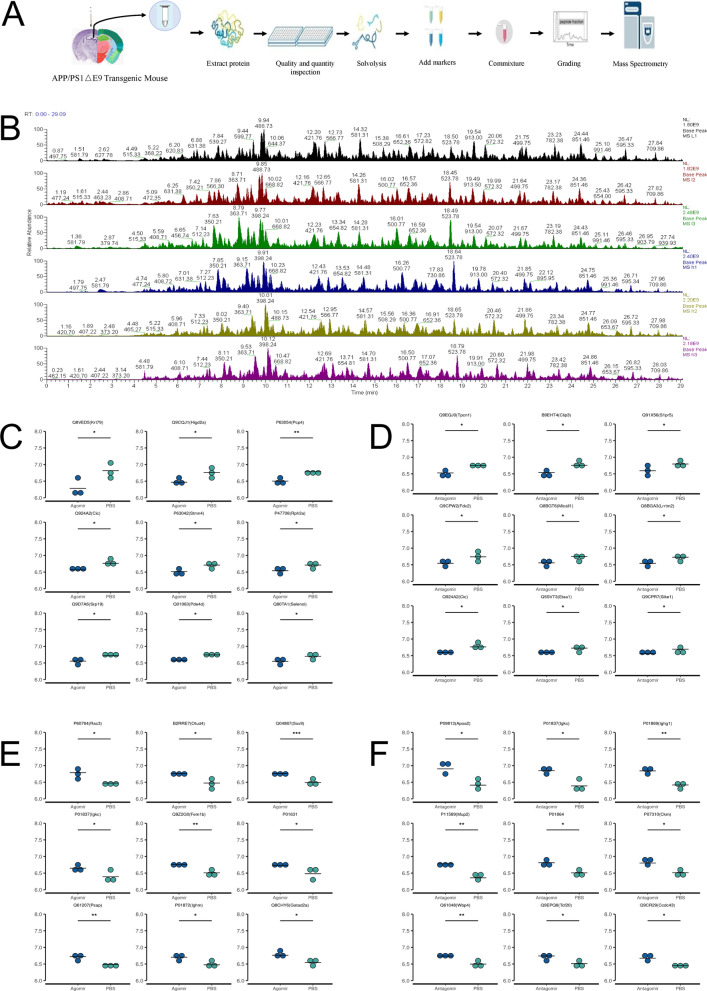
Results of proteomics analysis. **A** Operational flow chart of proteomics. **B** Base peak plot of extracted proteins. **C** Top nine proteins with decreased expression in agomir group compared with the PBS group. **D** Top nine proteins with increased expression in agomir group compared with the PBS group. **E** Top nine proteins with decreased expression in antagomir group compared with the PBS group. **F** Top nine proteins with increased expression in antagomir group compared with the PBS group. *P < 0.05

**Fig. 7:**
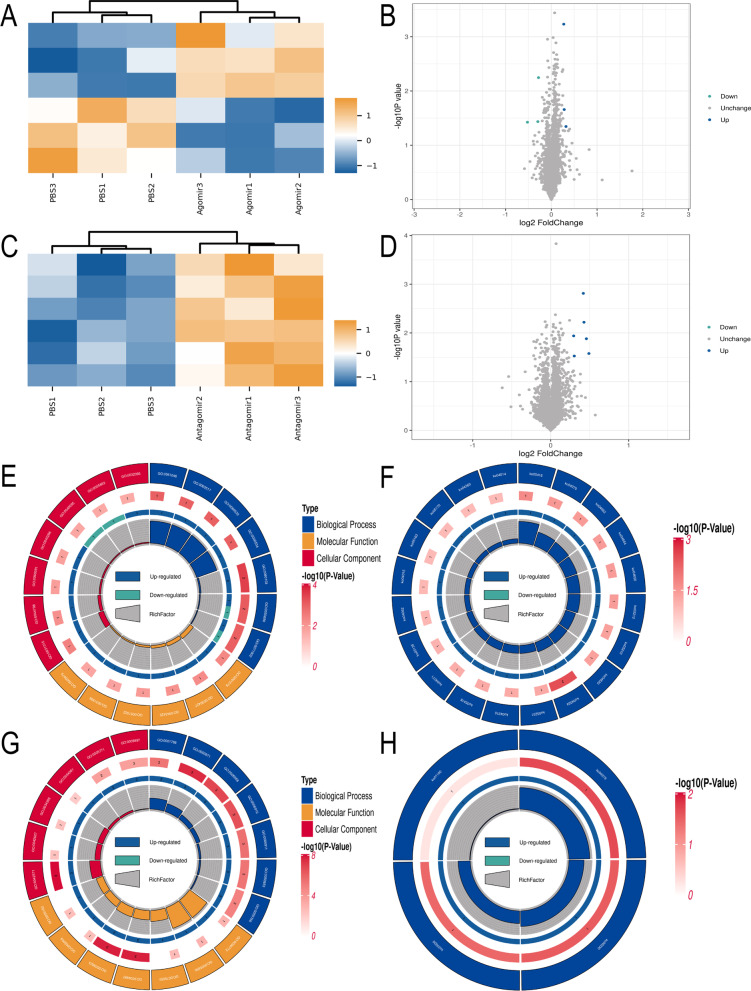
Results of KEGG and GO analyses of differentially expressed proteins (DEPs) identified by proteomics analysis. **A** Cluster analysis of DEPs between agomir group and PBS group. **B** Volcano plot of DEPs between agomir group and PBS group. **C** Cluster analysis of DEPs between antagomir group and PBS group. **D** Volcano plot of DEPs between antagomir group and PBS group. **E** GO analysis of DEPs between agomir group and PBS group. **F** KEGG analysis of DEPs between agomir group and PBS group. **G** GO analysis of DEPs between antagomir group and PBS group. **H** KEGG analysis of DEPs between antagomir group and PBS group

The hippocampus in each of the mice was collected for proteomics analysis (Fig. 6 and Additional file 1: Fig. S1; Additional file 12: Table S10). Compared with the PBS group, the top nine proteins with decreased expressions in the agomir group included Selenoi, Pde4d, Srp19, Rph3a, Stmn4, Cic, Pcp4, Higd2a, and Krt79, while the top nine proteins with increased expressions included Rac3, Otud4, Sox9, Igkc, Fem1b, P01631, Psap, Ighm, and Gatad2a. Compared with the PBS group, the top nine proteins showing increased expressions by antagomir were Apoa2, Igkc, Ighg1, Mup2, P01864, Ckm, Wbp4, Tcf20, and Ccdc43, whereas the top nine proteins with decreased expressions were Sike1, Etaa1, Cic, Lrrtm2, Micall1, Fdx2, S1pr5, Clip3, and Tpcn1. The volcano plots of the DEPs showed that compared with the PBS group, three proteins (i.e., Rac3, Otud4, and Sox9) were up-regulated and other three proteins (i.e., Pcp4, Higd2a, and Krt79) were down-regulated in the agomir group (Fig. 7B), while compared with PBS group, six proteins (i.e., Apoa2, Igkc, Ighg1, Mup2, P01864 and Ckm) were up-regulated in the antagomir group (Fig. 7D). The results of the KEGG and GO analyses of the DEPs are shown in Fig. 7 and Additional file 13: Table S11. Compared with the PBS group, the significantly enriched GO terms in agomir group included extrinsic component of mitochondrial outer membrane, nuclear transcription factor complex, kinocilium, and others, while the significantly enriched KEGG pathways were cAMP signaling pathway, viral myocarditis, VEGF signaling pathway, and others. In comparison with the PBS group, the enriched GO terms in the antagomir group were immunoglobulin complex, circulating, external side of plasma membrane, multivesicular body, among others, and the enriched KEGG pathways were cholesterol metabolism, arginine and proline metabolism, PPAR signaling pathway, and others.

### Sox9 as the target of miR-22-3p and involved in the NF-kB pathway

**Fig. 8:**
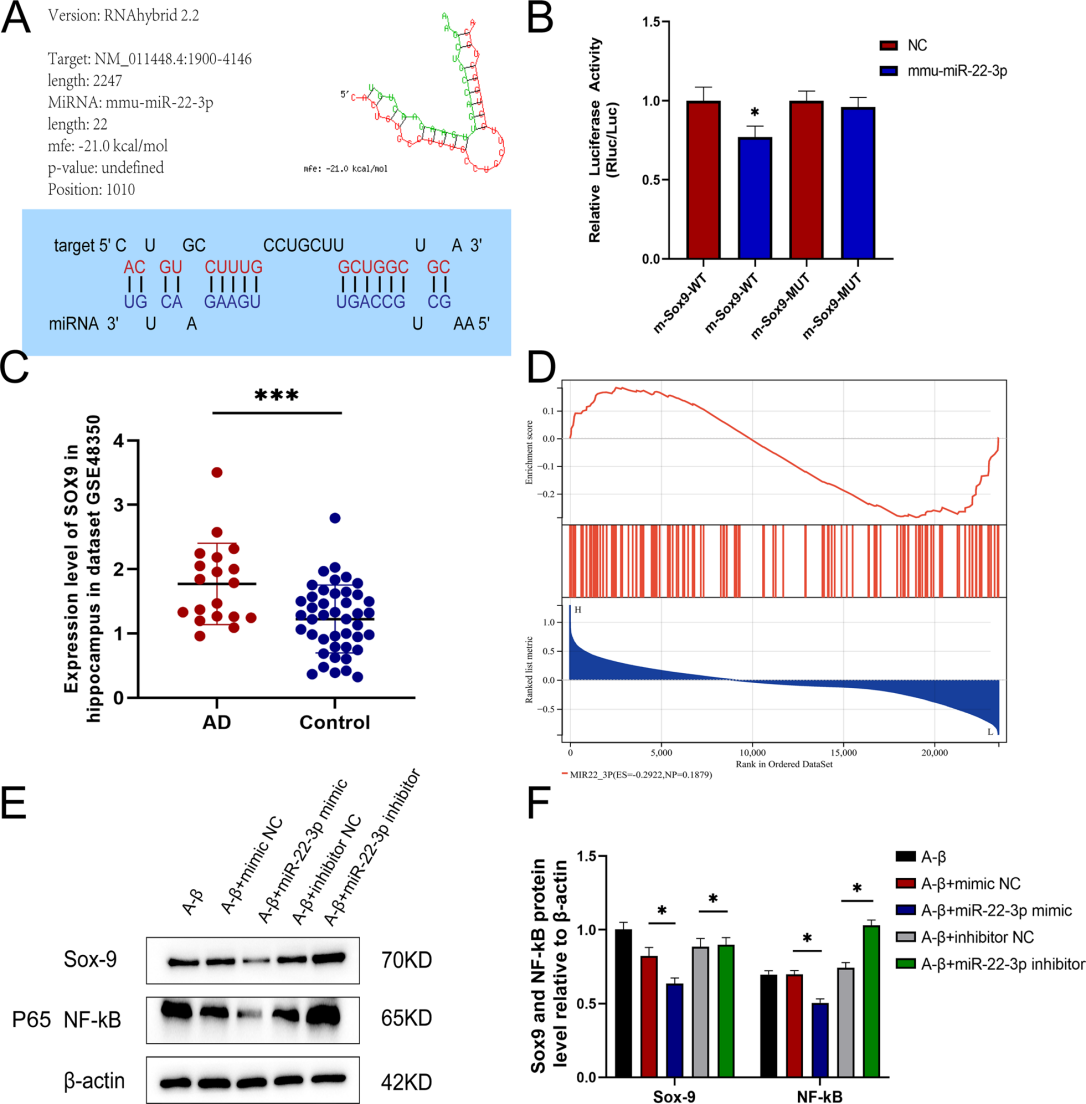
Results of luciferase reporter experiments. **A** Prediction of binding effect between miR-22-3p and Sox9 on the RNAhybrid site. **B** The luciferase assay shows that miR-22-3p causes a decrease in fluorescence in the wild group but not in the mutant group. **C** Sox9 expression is increased in AD brain tissue based on the GEO database. **D** Results of GSEA shows that miR-22-3p binds to more genes in the group with high expression of Sox9. **E** The expressions of Sox9 and NF-kB proteins are decreased as the level of miR-22-3p is elevated. **F** Statistics of western blot results. *P < 0.05, ***P < 0.005, n = 3

In order to confirm the binding effect of miR-22-3p, the binding targets of miR-22-3p were first predicted by RNAhybrid. The results revealed the minimum free energy of miR-22-3p and *Sox9* as − 21.0 kcal/mol, and the binding effect occurred at 17 positions out of 22 bases (Fig. 8A). The binding relationship between miR-22-3p and *Sox9* was further confirmed by the luciferase experiments. Specifically, the results showed that adding miR-22-3p to the wild *Sox9* group resulted in a significant decrease in fluorescence but not in the mutant *Sox9* group (Fig. 8B; Additional file 14: Table S12). Our results based on the dataset GSE48350 showed that the expression of *Sox9* was increased in the AD group (Fig. 8C). Based on the expression of *Sox9* in GSE48350, the AD patients were divided into two groups, i.e., the top half the high-expression group and the bottom half the low-expression group in the GSEA. More genes associated with miR-22-3p were identified in the high-expression group than those in the low-expression group (Fig. 8D). Based on the results of the proteomics analysis, the Sox9 with the smallest p value was selected for further verification to reveal that the Sox9 was closely related to the NF-kB pathway, as reported previously [33]. Therefore, the proteins in the NF-kB protein complex were also chosen for further verification. The results showed that the expressions of Sox9 and proteins in NF-kB complex were decreased when the expression of miR-22-3p was highly increased (Fig. 8E, F; Additional file 15: Table S13). Based on these results, it was proposed that the increased expression of miR-22-3p led to the decreased expression of Sox9 in the NF-kB pathway, the decreased expression of Bax, and the increased expression of Bcl-2, ultimately reducing the deposition of Aβ and alleviating the neural apoptosis.

## Discussion

Studies have demonstrated that the use or targeting of naturally occurring miRNAs represent a promising alternative to existing RNA-based therapies and may potentially enhance the therapeutic effects, compared with the synthetic siRNAs or ASOs that regulate only a single target gene [34, 35]. AD is a common disease regulated by polygenes, in particular, with miRNAs involved in the development and progress of AD [36, 37]. Our previous study showed that the expression of miR-22-3p was decreased in the blood of AD patients [15]. Therefore, we further explore the molecular mechanism underlying the involvement of miR-22-3p in the formation and development of AD.

In general, few AD patients have been given brain surgery in China, making it extremely challenging, if not impossible, to obtain sufficient brain tissues of AD patients. Therefore, the statistical analysis was performed based on the data derived from the GEO database to reveal the reduced expression of miR-22-3p in the brain tissue and blood of AD patients. These findings prompted our hypothesis that the increased level of miR-22-3p could improve AD. In order to explore the explicit functions of miR-22-3p, we identified the distribution of miR-22-3p and its association with nervous system disorders. Due to the lack of data of AD in the database, we analyzed other nervous diseases and found that the miR-22-3p was distributed in the nervous system broadly related to many other nervous system diseases, e.g., Parkinson’s disease and Huntington’s disease, implying its possible relationship with AD. Furthermore, we studied the role of miR-22-3p on Aβ-treated HT22 cells and found that miR-22-3p could improve the apoptosis of HT22 cells, indicating its function at cellular level.

As the critical area in brain for learning and memory, the hippocampus is especially vulnerable to damage in the early stages of AD [38]. In order to explore the role of miR-22-3p in-depth, the miR-22-3p was injected into the mouse hippocampus to show significant improvement of the memory and cognitive abilities in the mouse model of AD, as evaluated by the Morris water maze test [39]. It was noted that the Aβ deposition was decreased during this process, as shown by the immunohistochemical staining. However, no evident neuronal loss was observed based on the results of HE staining and Nissl staining on the hippocampus. Some researchers believed that, in addition to neuronal loss [40], both Aβ deposition and neurofibrillary tangles were also the main pathological symptoms of AD [41]. Furthermore, several studies have also found that the neuronal loss and Aβ deposition occasionally are not detected simultaneously [42].

Proteomics analysis based on the hippocampus was further performed to identify the target of miR-22-3p. Both KEGG and GO analyses of the DEPs identified by the proteomics investigation indicated that several pathways were significantly enriched, e.g., cAMP, viral myocarditis, and VEGF signaling pathways. The expressions of proteins involved in these pathways were further analyzed in AD patients based on the data derived from the public database. The results showed that the *P* value of protein Sox9 was the smallest, suggesting that Sox9 was the target by miR-22-3p. This was further verified by our luciferase experiments. Recent studies suggested that miR-22-3p also played an important role in modulating the differentiation and maturation of immune cells, showing that miR-22-3p was differently expressed in different types of immune cells [43], while it is well known that immunity is critical for AD [44]. Furthermore, studies suggested that miR-22-3p was a critical regulator of the differentiation of dendritic cells by targeting *Irf8*, which was highly expressed in the conventional dendritic cells [45], and the miR-22-3p could also directly up-regulate the expression of *Csf1r* in the final step of dendritic cell maturation [46]. *Sox9* is known as the initiator of gliogenesis, i.e., during early astrocyte differentiation, the transcription factor *Pitx1* promotes the astrocyte differentiation by regulating the *Sox9* gene [47]. Furthermore, it has been reported that Aβ can increase neurocan expression by regulating the expression of *Sox9* in astrocytes [48], suggesting that *Sox9* is closely related to Aβ. These findings indicated that miR-22-3p exerted a protective effect in AD by acting on *Sox9*, which was also confirmed by the results of western blot analysis in our study.

As a key mediator of brain inflammation in AD, the NF-κB is a family of redox-sensitive transcriptional factors, containing the binding sites for the promoter region of the genes involved in both amyloidogenesis and inflammation [49]. Furthermore, it has been reported that miRNAs target *Sox9* via the NF-κB signaling pathway [33]. Treatment of Ishikawa cells with TNF-a caused induction of Sox9 protein expression and increased p65 stability in nuclei. Transient transfection of p65 also caused an increase in endogenous Sox9 expression at mRNA and protein levels [50]. Based on the results of western blot analysis, the protein level of Sox9 was decreased, followed by the decline of the proteins in the NF-κB complex, confirming that miR-22-3p could ameliorate the AD by targeting *Sox9* through the NF-κB signaling pathway in the hippocampus.

## Conclusion

Our previous study revealed the low expression of miR-22-3p in AD patients, while our current study investigated the role of miR-22-3p in AD and found that high expression of miR-22-3p could alleviate cell apoptosis and improve cognition ability in AD mice. As the target of miR-22-3p identified by proteomics analysis, the binding effect of *Sox9* and miR-22-3p was also verified by the luciferase experiments. The Sox9 was further confirmed to be involved in the NF-κB signaling pathway. To conclude, microRNA-22-3p ameliorates AD by targeting Sox9 through the NF-κB signaling pathway in the hippocampus.

## Supplementary Information

**Additional file 1: Figure S1.** Proteomics quality control.

**Additional file 2: Figure S2.** The stability and toxicity of miRNAs.

**Additional file 3: Table S1.** Expression of miR-22-3p in AD brain tissue dataset GSE157239

**Additional file 4: Table S2.** Expression of miR-22-3p in AD blood dataset GSE120584.

**Additional file 5: Table S3.** Results of miR-22-3p in TCGA and SRA databases.

**Additional file 6: Table S4.** Flow cytometry results of blank group and Aβ modeling group.

**Additional file 7: Table S5.** Results of Western blotting of blank group and Aβ modeling group.

**Additional file 8: Table S6.** Flow cytometry results of miR-22-3p acting on AD cell model.

**Additional file 9: Table S7.** Western blotting results of miR-22-3p acting on AD cell model.

**Additional file 10: Table S8.** Results of water maze test in AD mice.

**Additional file 11: Table S9.** Aβ immunohistochemical staining results.

**Additional file 12: Table S10.** List of protein quantification and differential analysis.

**Additional file 13: Table S11.** KEGG and GO analysis of differential proteins.

**Additional file 14: Table S12.** Results of the luciferase assay.

**Additional file 15: Table S13.** Expression of Sox9 in the hippocampus in the GSE48350 dataset.

## Data Availability

The datasets generated and/or analyzed during the current study are available in the Gene Expression Omnibus (GEO) repository with the accessions of GSE157239, GSE120584, and GSE48350.

## Abbreviations

Aβ: Amyloid β
AD: Alzheimer’s disease
APP/PS1: APPswe/PSEN1dE9
GEO: Gene expression omnibus
miR-22-3p: MicroRNA-22-3p
miRNAs: MicroRNAs
NC: Normal control
SRA: Sequence read archive
sRNA-Seq: Small RNA-Seq
TCGA: The Cancer Genome Atlas
