# Supplementary figures and images for: MicroRNA-22-3p ameliorates Alzheimer’s disease by targeting SOX9 through the NF-κB signaling pathway in the hippocampus
Source: J Neuroinflammation. 2022 Jul 12;19:180. doi: 10.1186/s12974-022-02548-1 (PMC9277852; doi:10.1186/s12974-022-02548-1)

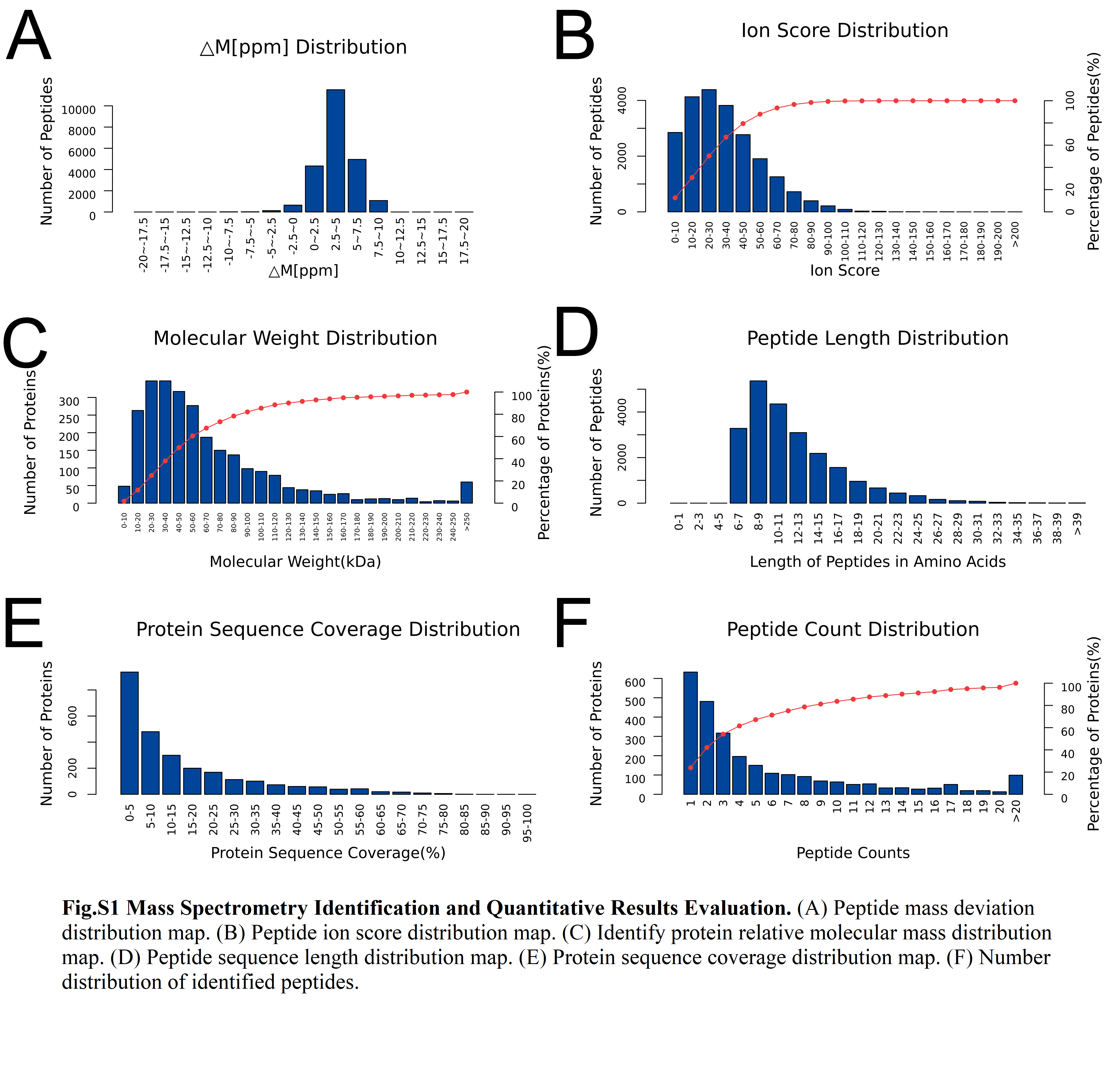

Supplement: Supplementary file 1 — Additional file 1: Figure S1. Proteomics quality control. [file 12974_2022_2548_MOESM1_ESM.tif]

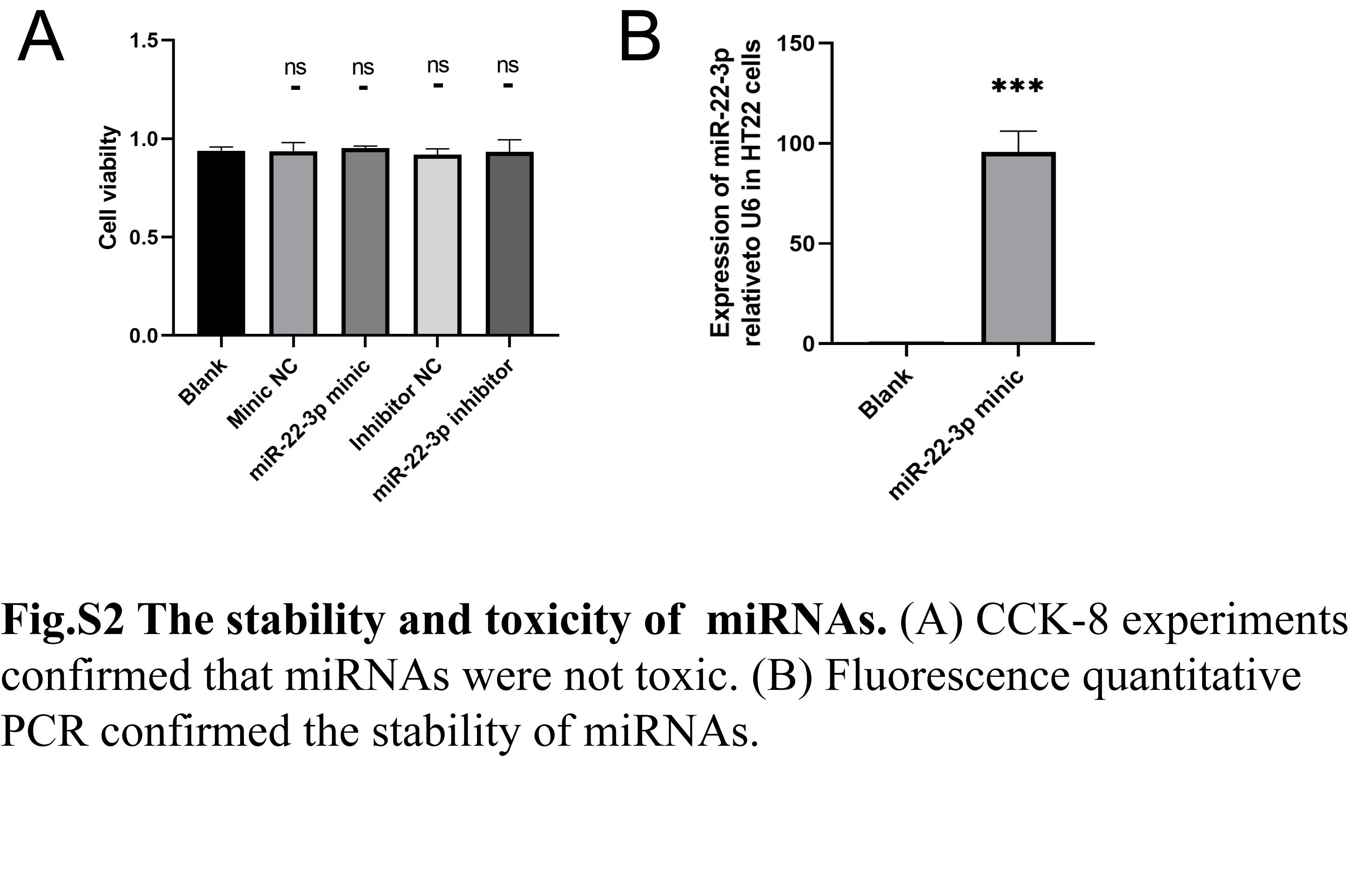

Supplement: Supplementary file 2 — Additional file 2: Figure S2. The stability and toxicity of miRNAs. [file 12974_2022_2548_MOESM2_ESM.tif]
